# Amino Acids Aided Sintering for the Formation of Highly Porous FeAl Intermetallic Alloys

**DOI:** 10.3390/ma10070746

**Published:** 2017-07-04

**Authors:** Krzysztof Karczewski, Wojciech J. Stepniowski, Marco Salerno

**Affiliations:** 1 Department of Advanced Materials and Technologies, Faculty of Advanced Technology and Chemistry, Military University of Technology, 00-908 Warszawa, Poland; krzysztof.karczewski@wat.edu.pl; 2 Materials Characterization Facility, Istituto Italiano di Tecnologia, 16163 Genoa, Italy; marco.salerno@iit.it

**Keywords:** intermetallics, sintering, powder metallurgy, FeAl, combustion, cysteine, phenyalanine, gas releasing agents

## Abstract

Fabrication of metallic foams by sintering metal powders mixed with thermally degradable compounds is of interest for numerous applications. Compounds releasing gaseous nitrogen, minimizing interactions between the formed gases and metallic foam by diluting other combustion products, were applied. Cysteine and phenylalanine, were used as gas releasing agents during the sintering of elemental Fe and Al powders in order to obtain metallic foams. Characterization was carried out by optical microscopy with image analysis, scanning electron microscopy with energy dispersive spectroscopy, and gas permeability tests. Porosity of the foams was up to 42 ± 3% and 46 ± 2% for sintering conducted with 5 wt % cysteine and phenylalanine, respectively. Chemical analyses of the formed foams revealed that the oxygen content was below 0.14 wt % and the carbon content was below 0.3 wt %. Therefore, no brittle phases could be formed that would spoil the mechanical stability of the FeAl intermetallic foams. The gas permeability tests revealed that only the foams formed in the presence of cysteine have enough interconnections between the pores, thanks to the improved air flow through the porous materials. The foams formed with cysteine can be applied as filters and industrial catalysts.

## 1. Introduction

Iron aluminides, including FeAl intermetallic alloys, are well-known materials for their remarkable mechanical properties and excellent corrosion resistance at room and elevated temperatures [1,2,3,4]. Thus, these materials are often used as protective coatings for exploitation in high temperature aggressive environments due to the formation of the protective oxide layer on their surface [5,6,7,8,9,10]. Also for this reason, application of FeAl intermetallic alloys can be a key for the development of filtrating elements in chimneys, where extremely hot outlet gasses are being ejected. To achieve this goal, surface area development of FeAl must be researched. Classical methods, like the melting of metal in foaming agents, are hard to apply for intermetallics due to their high melting points. Thus, alternative approaches must be worked out.

Powder metallurgy is one of the methods applied for fabrication of intermetallic components [11]. In the case of intermetallics, like FeAl, self-propagating high-temperature synthesis (SHS) occurs, locally enhancing the temperature during sintering, which is advantageous to the quality of the formed massive element [12,13,14]. To obtain porous materials with sintering, various approaches are being applied. One of the most popular approaches is the application of a leachable space holder, like NaCl, during sintering. After formation of the massive, porous element, the NaCl is leached out and porosity is revealed. Following this approach, metallic foams made of Al [15,16,17], Cu [18], FeAl [19], Mg [20,21], Ti [22], Ti_5_Si_3_ [23], TiAl [24], and TiAl_3_ [25] have been obtained. Nevertheless, using sodium chloride as the space holder may bring some risks since, when the leaching out process is too long, potentially galvanic cells may be formed and corrosion may occur. Moreover, not-fully leached out NaCl would also be detrimental for the same reason. Other materials, like poly methyl methacrylate (PMMA) and stearic acid, are sometimes used as the space holders [26]. However, other approaches have also been developed in order to obtain metallic foams. For example, Shi et al. reported the fabrication of porous Ti-Al alloy with thermal explosion (TE) reaction during the sintering [27]. Another approach that is easy to apply during sintering is in-situ gas release. One of the most explored techniques is the lost carbonate sintering (LCS). This technique employs carbonates and during sintering at high temperatures, the carbonate decomposes into metal oxide and carbon dioxide, where CO_2_ works as a foaming agent, according to the following reaction

(1) $${\overset{n}{X}}_{2}{\left(CO_{3}\right)}_{n}\overset{T}{\to }{\overset{n}{X}}_{2}O_{n}+nCO_{2}↑,$$

where *X* is the element and *n* is its valence.

Using this approach, compounds like K_2_CO_3_ [28,29,30], (NH_4_)_2_CO_3_ [31], and NH_4_HCO_3_ [31] were used and foams made of Cu [28,29,30] and Ti [31] were formed, respectively. The major disadvantage of the LCS approach is the formation of metal oxide when the metal carbonate is applied. Remaining basic oxide may be corrosive to the d-electronic metals, due to its amphoterism. On the other hand, Laptev et al. reported the application of ammonia carbonate and ammonium hydro-carbonate as gas releasing agents. Thus, the above-mentioned issue has been partially solved, because gaseous ammonia was released instead of metal oxide, enhancing the foaming performance of the carbonate [31]. However, the products of the combustion are not chemically ambient, and thus could go into reactions with the sintered metals.

Organic compound assisted sintering is currently being explored and provides chemically ambient products like carbon dioxide and water steam. During sintering of elemental powders, the added organic compounds—e.g., oxalic acid [32], eosin [33], stearic acid [34] or cholesteryl myristate [34]—combust to those compounds. Nonetheless, this approach also provides an issue that has to be solved: at low air (oxygen) admission, namely that the added organic does not combust to carbon dioxide but to carbon monoxide or simply to carbon. At the decomposition sites, much carbon is being found [32,33,34], which at elevated temperatures may result in the formation of brittle carbides, spoiling the performance of the intermetallic foam material. The carbon content may be minimized by thermal post-treatment, like annealing at high temperatures in air. An elegant alternative approach to solve this issue is the use of organic compounds decomposing with ambient gases like nitrogen.

In this work, the formation of FeAl intermetallic foams with the use of amino acids as gas releasing agents was studied. The main advantage of the proposed approach is the decomposition of the organic foaming agent with formation of nitrogen that is an ambient gas. The more nitrogen is formed, the lower the risk of secondary reactions (i.e., carburization) of the formed compounds with the sintered metals.

Decomposition of amino acids can eject nitrogen. Thus, an inert gas works as the foaming agent, decreasing the risk of carburization of the surface pores and formation of brittle carbides. In order to investigate this, two different amino acids were used as the additive, namely phenylalanine and cysteine. Phenylalanine is composed only of carbon, oxygen, nitrogen, and hydrogen and decomposes according to the following equation

(2) $$4C_{9}H_{11}NO_{2}+43O_{2}\overset{T}{\to }36CO_{2}↑+22H_{2}O↑+2N_{2}↑,$$

On the other hand, cysteine poses also sulfur and decomposes with sulfur dioxide release

(3) $$4C_{3}H_{7}NO_{2}SH+19O_{2}\overset{T}{\to }12CO_{2}↑+14H_{2}O↑+4N_{2}↑+4SO_{2}↑\;,$$

By adding cysteine to the Fe and Al powder elements, we are thus also able to check whether the formation of sulfur oxides may have any influence on the pore formation.

The major motivations of the use of the amino acids as gas releasing agents are the volume of the in situ produced gases (Table 1) and the formation of gaseous nitrogen, diluting remaining gaseous products including carbon dioxide, and thus decreasing the chemical interaction between the formed sinter and produced gas. Moreover, amino acids as well as other organic compounds [32,33,34] work as mentioned gas releasing agent, but first they act as space holders in the same way as e.g., NaCl [15,16,17,18,19,20,21,22,23,24,25].

Table 1 compares various organic compounds suitable to be applied as gas-releasing foaming agents. Chemical compounds used up to date release only carbon dioxide and water steam, as well as bromine [32,33,34] (Table 1). In this study we have selected amino acids that also form chemically inert nitrogen. However, the most important consideration is the foaming efficiency, namely the volume of formed gases estimated at ambient conditions from the combustion of 1 g of the additive. According to Table 1, cysteine (1.5 dm^3^/g) and phenylalanine (2.0 dm^3^/g) are much better options than the previously used oxalic acid and eosin Y (0.9 dm^3^/g), although significantly more gas is produced by palmitic acid combustion (3.0 dm^3^/g). Combustion of the latter additive, however, results in relatively large amounts of CO_2_.

## 2. Results and Discussion

**Figure 1:**
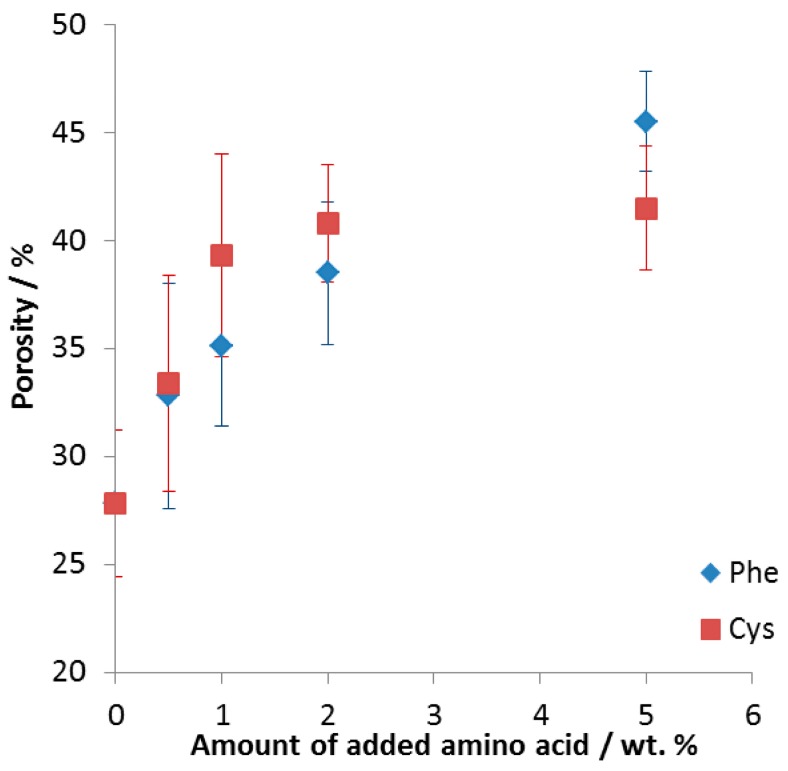
Porosity of the formed intermetallic FeAl foams as a function of the amount of amino acid added. Phe (blue diamonds): phenylalanine, Cys (red squares): cysteine.

In Figure 1, one can see that the addition of amino acids resulted in a significant increase of porosity of the FeAl intermetallic alloys: from 28 ± 3% when sintering without additive, up to 42 ± 3% and 46 ± 2% when adding 5 wt % of either cysteine or phenylalanine, respectively. The samples above 5 wt % were falling apart (see Figure S2).

**Figure 2:**
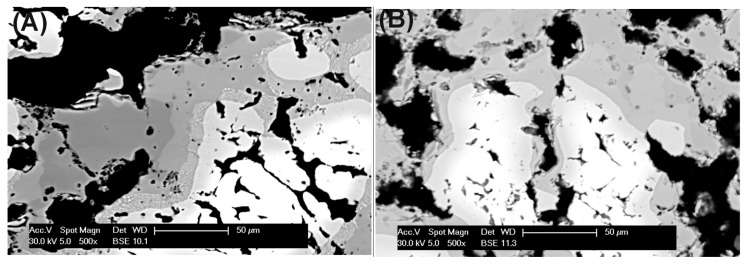
SEM images of metallic foams obtained after sintering of elemental Fe and Al powders with 5 wt % of (**A**) cysteine and (**B**) phenylalanine.

Apparently, differences in the morphology of the formed intermetallic sinters were also observed, as shown by the micrographs in Figure 2: larger pores, with size up to ~50 µm, are sometimes present in the sample formed with cysteine Figure 2A as compared to the pores formed with phenylalanine Figure 2B. For potential applications in filtration systems, the formation of pores is extremely useful, together with their interconnectivity, because it this increases the path length of the flowing gas. In the case of samples prepared with phenylalanine in Figure 2B, a larger amount of smaller pores can be noticed, likely with a high number of interconnections between them.

**Figure 3:**
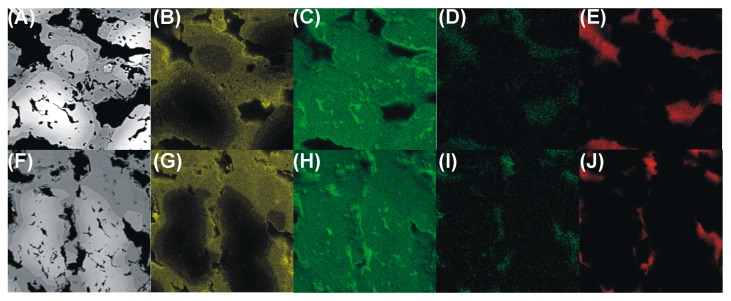
SEM images (**A**,**F**) and elemental EDS maps showing the distributions of aluminum (**B**,**G**), iron (**C**,**H**), oxygen (**D**,**I**), and carbon (**E**,**J**) in FeAl intermetallic foams formed via cysteine (**A**–**E**) and phenylalanine (**F**–**J**) assisted sintering.

Elemental maps of the formed intermetallic foams are shown in Figure 3, from which it appears that there are only traces of oxygen in the formed samples Figure 3D,I. This shows that no brittle oxides were formed, which would be detrimental to the mechanical stability of the foams.

Moreover, due to the application of amino acids, a considerable amount of nitrogen gas was formed during the sintering process, and consequently also a low amount of carbon was found at the pore bottoms of the foams Figure 3E,J. This is advantageous because at elevated temperature the system cannot form brittle carbides either, thanks to the lack of carbon. The qualitative picture given in the images of Figure 3 is confirmed in the results of quantitative EDS analyses reported in Table 2.

According to them, the concentration of oxygen and carbon in the formed foams is relatively small. In particular, oxygen is only present in 0.11 ± 0.04 wt % and 0.14 ± 0.06 wt % for the foams formed with 5 wt % cysteine and phenylalanine, respectively. One has to be aware that during sintering and chemical combustion of the added amino acids, access to the oxygen may be limited and carbon monoxide or carbon may be formed instead of carbon dioxide. This would significantly contaminate the surface with carbon and allow carburization to occur during the process. Another issue is the oxidation of aluminum during the sintering. As one can notice from the elemental analyses, thanks to the use of amino acids those effects have been minimized, likely because formed nitrogen is allowed to dilute and leach out the formed gases, so their interaction with the foam was significantly shortened. According to Table 1, cysteine has a much higher number ratio of nitrogen atoms to those of other elements in the molecule, thus much more inert gas is formed during the sintering than is the case with phenylalanine.

The morphology analyses of the foams in the core of the material, summarized in Table 3, reveals that the average pore diameter of the foams is ~30 µm in both cases of phenylalanine and cysteine additive. However, the standard deviations of the pore diameters are very large, confirming their broad distribution. Analysis of the sub-surface zones reveals small differences: the foams formed with cysteine have ECD values of the pores comparable to those in the core of the sample, as well as the foams formed with phenylalanine having comparable mean pore size close to the surface (~25 µm). The ECD of pores of reference material formed without additive is comparable, however the number of pores (porosity) is greater.

**Figure 4:**
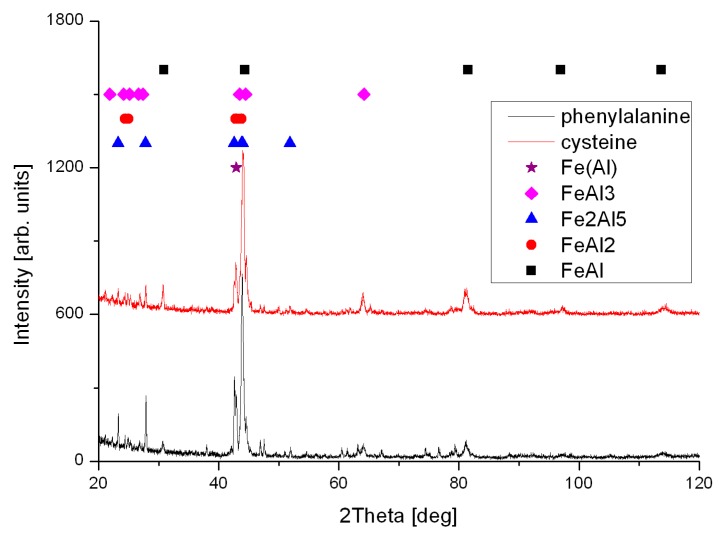
XRD patterns of samples formed by sintering of elemental powders with additive of 5 wt % of cysteine and phenylalanine.

The foams have been made by sintering of elemental powders, however self-propagating high-temperature synthesis enabled the formation intermetallic phases, where FeAl intermetallic phase is a dominant Figure 4. Therefore, such obtained foams can be suitable for exploitation in corrosive and high-temperature environments, like catalysts, or industrial filters in chimneys.

**Figure 5:**
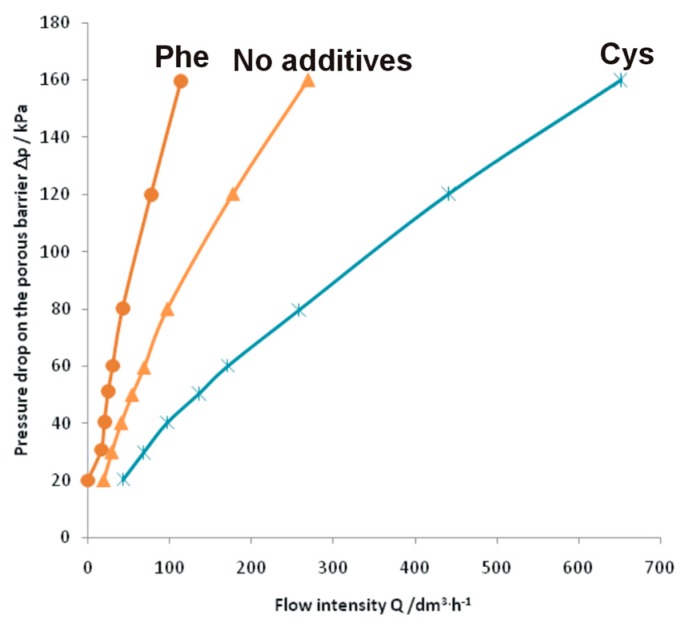
Relationship between air pressure drop and flow intensity during penetration through the porous FeAl intermetallic foam with (Phe for phenylalanine and Cys for cysteine) and without additives.

To examine whether the formed intermetallic foams may be used in filtering systems, permeability experiments were performed, which are presented in Figure 5. 

Surprisingly, compared to the foam formed without any additives [34], the foam formed with the use of phenylalanine showed a much greater flow resistance during the permeability experiments. With an overpressure of 20 kPa applied there was no air flow through the foam, meaning that pore interconnection in the samples was not appropriate for the devised application. Thus, metallic foams formed by sintering with phenylalanine cannot be used in gas filtering systems. This limitation is probably caused by the inhomogeneity in the distribution of the pores. Larger and bigger pores are likely found only locally and the interconnections among pores are absent. On the other hand, FeAl intermetallic foam formed with the use of 5 wt % cysteine showed much lower flow resistance than the foams formed without any additive. Therefore, this intermetallic foam is a promising material in filtering systems due to the specific porous morphology, the low residual carbon and oxygen content, and high-temperature resistance (FeAl intermetallic alloy). 

In summary, FeAl intermetallic foams were formed via amino acid assisted sintering of elemental powders. Analysis of the samples allowed us to draw the following conclusions:

- Intermetallic foams were formed with porosity up to 42 ± 3% and 46 ± 2% for 5 wt % addition of cysteine and phenylalanine, respectively.
- Only traces of carbon and oxygen were noticed after the sintering, which allows us to conclude that gaseous nitrogen formed during the process made the leaching out of the combustion products easier.
- Only the intermetallic foams formed with the use of cysteine showed improved gas permeability, likely provided by numerous pore interconnections and thus enlarged path for the flowing gases, making them possible candidate materials for use as porous gas filters.

## 3. Materials and Methods

The starting materials used in the research were: 99.9% purity Fe powder (average diameter of particle ~100 µm, NC 100.24/99.7%, ABCR GmbH & Co KG, Karlsruhe, Germany) and 99% purity Al powder (particle size <75 µm, AG 30–100/99.7%, BENDA–LUTZ SKAWINA, Skawina, Poland) (see Figure S1 for SEM images of the particles; and p.a. glycine and p.a. phenylalanine (all by POCh, Gliwice, Poland). Various amounts of amino acids, cysteine (Cys) or phenyalanine (Phe), were added to the reference composition (RC) in order to study the influence of the additive on the porosity. The RC was Fe-45Al (at %) and following mixtures were prepared: RC + 0.5 wt % Cys or PHe, RC + 1 wt % Cys or Phe, RC + 2 wt % Cys or Phe, and RC + 5 wt % Cys or Phe. Then, the powder mixtures were consolidated by uniaxial cold pressing under 700 MPa pressure into cylindrical 25-mm diameter and 6-mm high pellets. The sintering was carried out in the volume controlled environmental reactor, as previously reported [34], for 3 h at 700 °C in Ar atmosphere.

Porosity of the sinters was estimated from optical micrographs obtained with a Nikon MA200 optical microscope integrated with NIS-Elements software (both from Advanced Research Nikon, Shinagawa-Tokyo, Japan). To quantify the pore sizes of the formed FeAl intermetallic foams, optical microscopy and NIS-Elements software were used to obtain Equivalent Circle Diameter (ECD) of the pores. The ECD was estimated from the following equation [35]

(4) $$ECD=2\sqrt{\frac{A}{\pi }}$$

where *A* is the surface area of the analyzed grain.

Scanning Electron Microscopy (SEM) and energy-dispersive X-ray spectroscopy (EDS) analyses were done using a QUANTA 3D FEG scanning microscope (Philips, Amsterdam, the Netherlands) integrated with the X-ray DX4i/EDAX microanalysis device.

The permeability examinations were performed using a lab-made equipment. The samples for the examinations were circular with 25 mm diameter and 10 mm thickness. The samples were placed into a steel tube to seal the system from the side. Synthetic air was used as the working gas. The pressure drop on the sample was used as a direct measure of the permeability. Permeability measurements were conducted thrice for each sample.

## Figures and Tables

**Table 1:** Comparison of added organic compounds as gas release foaming agents.

Name, Formula, and Molar Mass	Structural Formula	Produced Gases	Produced Gas Volume ^1^/dm^3^	Reference
Cysteine, C_3_H_7_NO_2_S, 121.16 g/mol	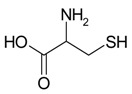	CO_2_, H_2_O, N_2_, SO_2_	1.48	This work
Phenylalanine, C_9_H_11_NO_2_, 165.19 g/mol	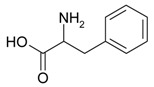	CO_2_, H_2_O, N_2_	2.03	This work
Eosin Y, C_20_H_6_Br_4_O_5_^2−^2Na^+^, 647.89 g/mol	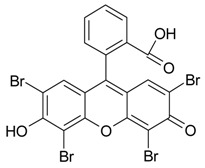	CO_2_, H_2_O, Br_2_	0.9	[33]
Crystalline oxalic acid, H_2_C_2_O_4_·2H_2_O, 126.07 g/mol	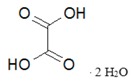	CO_2_, H_2_O	0.9	[32]
Palmitic acid, C_15_H_31_COOH, 256.42 g/mol	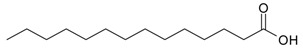	CO_2_, H_2_O	2.98	[34]

^1^ from combustion at ambient conditions of 1 g.

**Table 2:** Light elements EDS analyses of the porous FeAl intermetallic foams formed via sintering of Fe and Al elemental powders with 5 wt % of foaming agent.

Foaming Agent	Carbon Content		Oxygen Content	
	wt %	at %	wt %	at %
Cysteine	0.2 ± 0.1	0.9 ± 0.1	0.3 ± 0.1	1.1 ± 0.6
Phenylalanine	0.11 ± 0.04	0.3 ± 0.1	0.14 ± 0.06	0.4 ± 0.2

**Table 3:** Equivalent circle diameter of the pores of the FeAl intermetallic foams formed via elemental powder sintering with 5 wt % of amino acid foaming agent.

Foaming Agent	ECD/µm	
	In the Sample Core	At the sample surface
Without additive	28 ± 20	30 ± 17
Cysteine	29 ± 15	28 ± 16
Phenylalanine	32 ± 15	25 ± 11

## Supplementary Materials

The following are available online at www.mdpi.com/1996-1944/10/7/746/s1. Figure S1: Iron (left) and Aluminum (right) powders used as starting materials, Figure S2: Images of samples decomposed during sintering when too much additive was used.
