# Supplementary figures and images for: Amino Acids Aided Sintering for the Formation of Highly Porous FeAl Intermetallic Alloys
Source: Materials (Basel). 2017 Jul 4;10(7):746. doi: 10.3390/ma10070746 (PMC5551789; doi:10.3390/ma10070746)

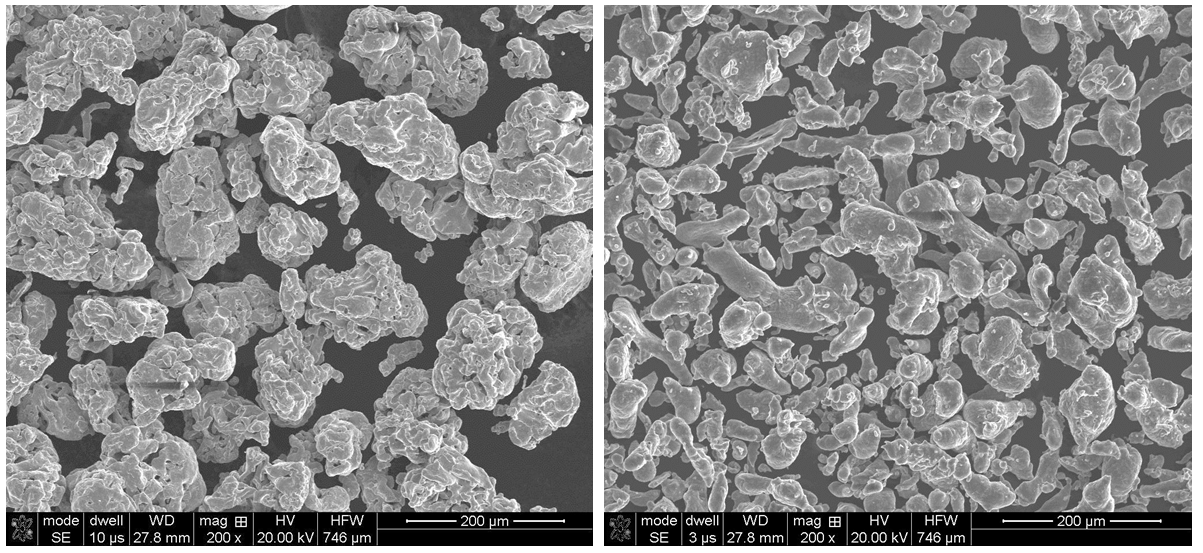

Supplement: Supplementary file 1 [file materials-10-00746-s001.zip › Fig.S1.bmp]

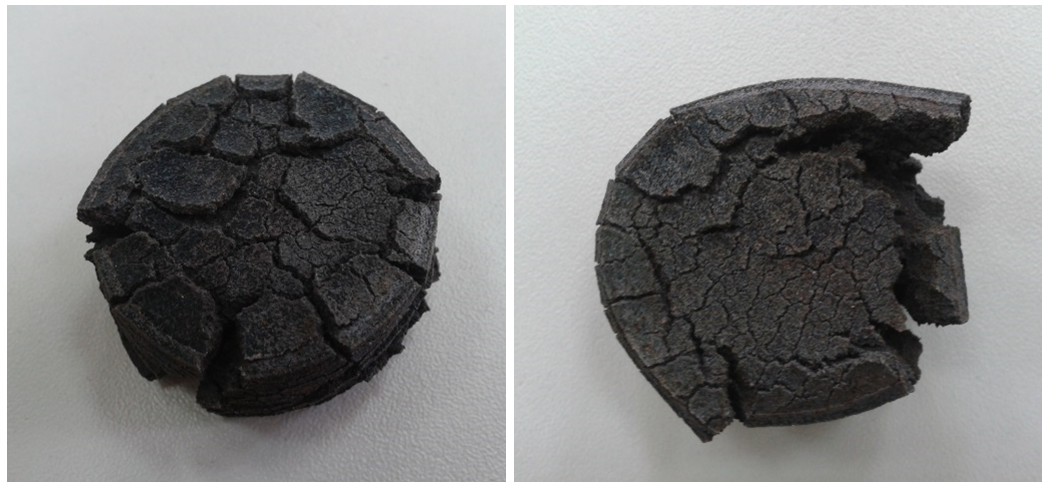

Supplement: Supplementary file 1 [file materials-10-00746-s001.zip › Fig.S2.bmp]
